# Effects of Ultrasound-Assisted Soy Lecithin Addition on Rehydration Behavior and Physical Properties of Egg White Protein Powder

**DOI:** 10.3390/foods13142252

**Published:** 2024-07-17

**Authors:** Sijia Cao, Xuanting Liu, Zhiyuan Zheng, Zhaohui Yan, Ting Zhang, Jingbo Liu, Ting Yu

**Affiliations:** 1 Jilin Provincial Key Laboratory of Nutrition and Functional Food, College of Food Science and Engineering, Jilin University, Changchun 130062, China; 15143692581@163.com (S.C.); lxt920523@163.com (X.L.); ohzzy20050612@163.com (Z.Z.); yzh19991019@163.com (Z.Y.); tingzhang@jlu.edu.cn (T.Z.); ljb168@sohu.com (J.L.); 2 Department of Nutrition, The Second Hospital of Jilin University, Changchun 130041, China

**Keywords:** ultrasound, egg white protein powder (EWPP), physicochemical properties, wettability, rehydration behavior

## Abstract

This study investigated the effects of ultrasound-assisted soybean lecithin (SL) on the rehydration behavior and physical properties of egg white protein powder (EWPP) and its ability to enhance the efficacy of EWPP instant solubility. The results of rehydration, including wettability and dispersibility, indicated that ultrasound (200 W)-assisted SL (5 g/L) addition had the shortest wetting time and dispersion time, which were 307.14 ± 7.00 s and 20.95 ± 2.27 s, respectively. In terms of powder properties, the EWPP with added SL had lower lightness, moisture content and bulk density. In addition, the increase in average particle size, net negative charge, free sulfhydryl group content and surface hydrophobicity indicated that ultrasound treatment facilitated the protein structures unfolding and promoted the formation of SL-EWP complexes. Overall, our study provided a new perspective for the food industry regarding using ultrasound technology to produce instant EWPP with higher biological activity and more complete nutritional value.

## 1. Introduction

Raw and processed eggs are extensively used in the food industry for their ideal nutritional and functional characteristics. Considering pathogen contamination and the difficulty of transportation and storage, liquid egg materials are usually dried into powder. With excellent processing properties, bioactivity and biocompatibility, EWPP plays an increasingly important role in food production, health care and biomaterials [1]. However, EWPP is often limited by wall hanging, poor dispersion and low solubility during rehydration, which remains a major challenge for its nutritional and processing performance [2]. For these problems, the novel united treatment to effectively enhance the rehydration capacity of EWPP has received attention as one of the bases for realizing various applications of egg white protein (EWP) [3,4].

Soybean lecithin (SL) is a natural emulsifier and surfactant, non-toxic, non-irritant, and easily degradable, and it has an asymmetric structure consisting of hydrophobic alkyl side chains and hydrophilic groups, which is an effective strategy to enhance the solubility of EWPP. In recent years, SL has been allowed to be applied to obtain nutritional-rich value protein and functional performance instant powder as the dispersant and wetting agent. Ji et al. [5] reported that coating SL on the whey protein isolate powder through a fluidized bed could increase the protein powder specific surface area and roughness, thereby improving its rehydration. In addition, Lallbeeharry et al. [6] added SL to whole milk before spray drying and observed hydrophilic shell formation and smaller contact angles of milk droplets. However, natural SL tends to be poorly dispersed in aqueous phase systems and has a limited effect as a surfactant. Furthermore, as the addition of surfactants before drying is not sufficiently effective, in actual industrial production, the use of SL to treat EWPP is not the preferred method. Since the co-drying approach possesses lower costs and higher efficiency [6,7], it is of interest to further investigate the co-drying of SL with other processing methods for the application of EWP.

Ultrasound (US) has been recognized as a safe, non-invasive and environmentally friendly technology in recent years, which has received extensive attention by affecting the structural properties of proteins and improving their functional and physicochemical properties [8]. It has been shown that the cavitation effect of US promotes the dissociation of protein aggregates, thereby facilitating the reaction of proteins with other molecules and enhancing their functions [9]. Therefore, US might improve the surface properties of EWPP by increasing the modification degree of SL to EWPP. In this purpose, we have conducted further research on US-assisted SL addition to obtain instant EWPP.

Overall, this work aimed to investigate the effects of US-assisted SL addition on the EWPP rehydration behavior and physical properties. The wettability and dispersibility of protein power were performed by wetting and dispersion time. Meanwhile, to further explore the powder characteristics, the color, moisture content, and bulk density of EWPP were studied. Finally, the mechanism of US-assisted SL enhancing the rehydration of EWPP was explained at the molecular level. By improving the rehydration performance of EWPP and the internal mechanism of its instant solubility, this study broke through the limitations of EWPP as raw materials in the food field, expanded its application as a food ingredient in the food industry, and provided a theoretical basis and a new attempt for the preparation of instant EWPP in the food processing industry.

## 2. Materials and Methods

### 2.1. Materials and Reagents

Fresh liquid eggs were purchased from DA RUN FA Supermarket (Changchun, China). EWPP (containing 86.5% protein) and SL (l-α-phosphatidylcholine, L8050) powder were purchased from Sigma Chemical Co., (St. Louis, Mo, USA). Other reagents were from Sinopharm Chemical Reagent Co., Ltd. (Shanghai, China). All reagents are analytically pure.

### 2.2. Preparation of Samples

SL at concentrations of 1 g/L, 3 g/L, and 5 g/L was added to the liquid proteins, and the pH was adjusted to 7.0 with 0.1 mol/L HCl or NaOH solution [8]. The solution was then homogenized under high pressure at 7000 rpm for 120 s (T 25 digital Ultra-Turrax, IKA, Staufen im Breisgau, Germany). The sample solution was then placed in an ice water bath and processed using an ultrasonic processor for 15 min (5 s on, 1 s off) to improve the homogeneity and stability. The ultrasonic processor model used was Sonics VCX 750 (Sonics Inc., Newtown, CT, USA) with an operating frequency of 20 kHz and power settings of 0 W, 200 W and 400 W. Finally, the samples were rapidly frozen to −80 °C and then dried under vacuum for further analysis.

### 2.3. Analysis of Powder Rehydration

#### 2.3.1. Wettability

The wettability assessment followed the approach of Tian et al. [10] with minor adjustments. A funnel was positioned vertically above a beaker containing 100 mL of deionized water at 25 ± 2 °C with the funnel’s outlet set 5 cm above the water surface. A mortar and pestle were placed inside the funnel, and the funnel’s mouth was obstructed. Subsequently, a precision measure of 1 g of sample was sprinkled around the mortar and pestle. The sample was then evenly and swiftly (within 2 s) dispersed into the deionized water. The wetting time was recorded under static conditions, which was defined as the duration from the powder’s initial contact with the air–water interface until it was fully immersed in the water. Each measurement was performed in triplicate at least.

#### 2.3.2. Dispersibility

The sample dispersion measurement was carried out by the method of Zhang et al. [11]. The sample (0.5 g) was added to a 100 mL beaker containing 50 mL of deionized water (25 ± 1 °C) and stirred on a magnetic mixer at 200 rpm. Dispersibility was the time from the start of mixing the powder to complete dissolution, and it was repeated 3 times.

#### 2.3.3. Contact Angle

The liquid properties of the samples were measured using a contact angle analyzer (OCA 15EC, Kruse, Germany) with a slight modification [12]. All the sample powder was extruded film by a manual press (Specac Ltd., Orpington, UK) for 10 s at 78.4 Mpa to form a pressed material with a diameter of 10 mm. Subsequently, 3 μL drops of deionized water were placed onto the press surface.

### 2.4. Powder Characteristics

#### 2.4.1. Color

According to the method of Masum et al. [13], the color parameters (L*, a* and b*) of powders were measured in triplicate using a Chroma meter (Konica Minolta Sensing Inc., Tokyo, Japan), where ‘L*’ was used to denote lightness, ‘a*’ to redness and greenness, and ‘b*’ to yellowness and blueness.

#### 2.4.2. Moisture Content

The determination of moisture content was referring to the method of Erdem and Kaya [14] with a few modifications. Samples of 1 g were set in an oven heated at 105 °C. The measurement was stopped when the average weight loss was less than 1 mg every 90 s. Moisture content was expressed as a percentage of moisture to the total weight of the sample.

#### 2.4.3. Bulk Density

The bulk density (ρ_bulk_) was investigated according the procedure described by Kapoor and Feng [15] with slight modifications. Briefly, 1 g of sample was manually dispensed into a 10 mL graduated cylinder. The ρ_bulk_ was calculated as shown in Equation (1):

(1) ρbulk=MV

ρ_bulk_ is the bulk density. *M* is the quality of the sample, while *V* is the volume occupied by the sample.

### 2.5. Scanning Electron Microscopy (SEM)

The differently treated samples were fixed on a stage with gold spray. The micromorphology was examined with a high-resolution Regulus 8100 scanning electron microscope (Hitachi, Tokyo, Japan) [16].

### 2.6. Zeta Potential and Particle Size

The particle size analyzer (Mastersizer 3000, Worcestershire, UK) was used to measure the zeta potential and particle size. EWPPs were dissolved to 1 mg/mL in deionized water, and 1 mL of dispersion was transferred into the measuring cell (10 mm × 10 mm × 45 mm). The cells were then stabilized for 120 s [17]. Each measurement was performed at 25 °C and repeated 3 times.

### 2.7. Measurement of Free Sulfhydryl (-SH) Group

The sample free sulfhydryl (-SH) group contents were determined using Shimada and Cheftel’s method [18] with a slight modification. By dissolving 4 mg of 5, 5-dithio-bis 2-nitrobenzoic acid (DTNB) in 1 mL of Tris-glycine buffer, Ellman’s reagent was prepared. Every sample (1 mL) added 125 µL of Ellman’s reagent and mixed in 2 mL Tris-glycine buffer. An ultraviolet-visible spectrophotometer (UV-2550, Shimadzu, Tokyo, Japan) was used to analyze the content of the free -SH group at 412 nm, and a buffer solution was used instead of the protein solution as a blank. The free -SH groups were calculated as shown in Equation (2):

(2) Free -SH (μmol/g)=(75.53 × A × D)/C

A is the absorbance at 412 nm; C is the concentration of the sample (mg/mL); D is the dilution factor.

### 2.8. Surface Hydrophobicity (H_0_) Measurement

The surface hydrophobicity of samples were measured according to the method proposed by Zhang et al. [19] by a fluorescence spectrophotometer (F-7100, Hitachi Co., Japan). Briefly, 4 mL of EWPP solution (0.1 mg/mL) was well mixed with 20 μL 8 mM ANS solution (10 mM phosphate buffer, pH 7.0) and then incubated for 15 min at room temperature in the dark. Relative fluorescence intensity was measured to express as *H*_0_ at the excitation wavelength of 390 nm with an emission spectra collection range of 400–600 nm and slit widths of 2.5 mm.

### 2.9. Apparent Viscosity

Dynamic rheological characterization of the samples was performed using a Discovery HR-1 rheometer (TA Instruments, New Castle, DE, USA) with a parallel plate geometry of 40 mm diameter at 25 °C. Samples were placed on a base plate with a gap (1000 μm), and excess material was removed with a scraper. A volume of 1.4 mL of each sample was applied to the lower plate and allowed to equilibrate for 1 min before analysis. The temperature was maintained at a steady 25 °C, and viscosity changes were recorded across shear rates from 1 to 300 s^−1^ [20,21].

### 2.10. Fourier Transform Infrared Spectroscopy (FT-IR)

The FT-IR measurement was performed according to the work of [20] with slight modifications. Approximately 200 mg KBr (died at 500 °C for 3 h) was mixed with 2 mg of EWPP and then pressed into a flake under an incandescent lamp. The FT-IR spectra of the samples were collected at wavenumbers of 4000–400 cm^−1^ using an FT-IR spectrophotometer (Shimadzu, Japan), and the background spectra were obtained with KBr flakes.

### 2.11. Differential Scanning Calorimetry (DSC)

The thermodynamic changes of the powder samples were analyzed using a Differential Scanning Calorimeter DSC7 (TA instruments, New Castle, DE, USA). The samples were heated from 30 to 250 °C in a sealed, fully enclosed aluminum pan at a ramp rate of 5 °C/min with a nitrogen flow rate of 50 mL/min. All experiments were carried out in triplicate.

### 2.12. Statistical Analysis

All experiments were conducted three times, and the data were expressed as mean ± standard deviation (SD). Statistical analysis was performed using Duncan’s new multiple range experiment and regression analysis.

## 3. Results and Discussion

### 3.1. Wettability and Dispersibility

**Figure 1:**
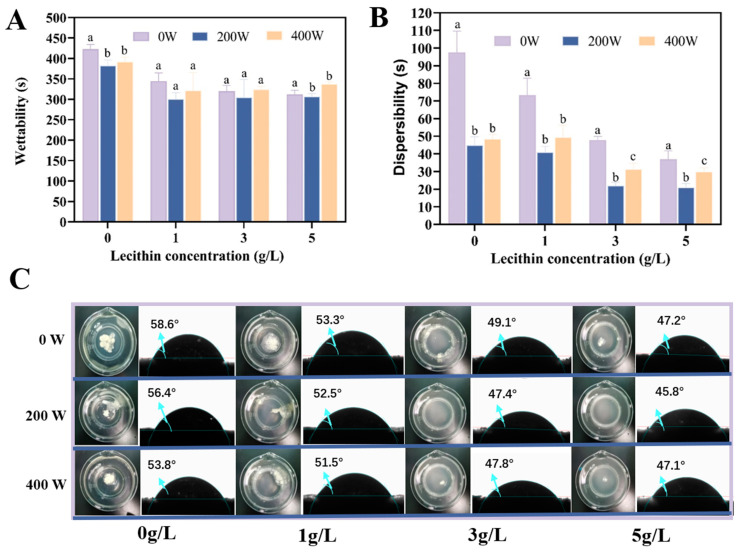
Effects of US-SL treatment on the wettability and dispersibility of EWPP. (**A**) Wettability, (**B**) dispersibility, (**C**) macroscopic photographs of EWPP after dispersing for 20 s, and the EWPP–water–wall contact angle, θ, were identified. Values that do not bear the same letter are significantly different (*p* < 0.05), and error bars represent the standard deviations.

We analyzed the effect of US-assisted SL addition on the wettability of EWPP, and the results of wetting time are shown in Figure 1A. It was shown that as the US power increased, the wetting time reduced from 422.78 ± 11.21 s to 307.14 ± 7.58 s (200 W), and then slightly increased to 337.57 ± 5.39 s (400 W), which indicated that the wettability of EWPP was promoted with 200 W US power treatment. The result above is perhaps due to the low-powered US being beneficial to promote the protein structure unfolding and the binding to SL. However, high-powered US power treatment would result in a slight aggregation of protein molecules, which prevented the formation of the SL-EWP complex [22]. In addition, it was noticed that the wetting time decreased as the SL concentration increased, which was consistent with our previous findings that SL enhanced the hydrophilicity of the EWP surface [12]. In summary, at a specific proportion, the SL-EWP complex can greatly reduce the wetting time of EWPP.

Dispersion of the powder, meanwhile, occurs almost simultaneously with wetting and was affected by the wetting step, so the dispersibility of EWPP with different ultrasonic power and SL concentration was further investigated. As shown in Figure 1B, the dispersibility of all samples (US or non-US) was positively correlated with SL concentration, which was attributed to high concentrations of SL providing more molecules that interact with the egg white protein. Notably, samples at any SL concentration achieved the shortest dispersion times at 200 W. These data suggested that moderate US treatment could effectively accelerate the diffusion of powder in the aqueous phase, which was similar to the findings in previous studies [23]. The dispersion time of EWPP was approximately 4.8 times lower than that of the control (ranging from 97.57 ± 12.07 s to 20.97 ± 20.27 s) at US 200 W-5 g/L SL. Moderate ultrasonic treatment accelerated the interaction of EWP and SL, which improved the hydrophilicity of EWP. The wetting and dispersion time of particles was shortened [3]. Similarly, the dispersion velocity decreased slightly under the high US of 400 W.

To further visualize the rehydration behavior of US-assisted SL addition on EWPP, Figure 1C showed the macroscopic photographs of EWPP after 20 s of dispersion as well as the contact angle of the EWPP surface at different US power and SL concentrations. The initial EWPP was apparently agglomerated compared to the US-treated samples. In addition, the powder was optimally dispersed at all SL concentrations under 200 W US treatment and almost completely dissolved at the addition of 5 g/L SL, with a significant decrease in contact angle from the initial 53.3° to 45.8°, which confirmed that US and SL treatment had a significant improvement on the solubility of EWPP.

### 3.2. Powder Property Analysis

The influence on US-assisted SL addition on the physical properties of EWPP are shown in Table 1. The color of the powdered products is a crucial sensory parameter that determines their acceptability and suitability for consumption. The powder showed lower lightness (L*) and higher redness (a*) values with the increase in SL concentration, which was probably affected by the color of SL itself. Meanwhile, the color parameter value of EWPP showed smaller changes with the increase in SL concentration after US. According to Zhou et al. [24], US treatment would cause the unfolding of the protein structure, which increased the exposed area of the molecules embedded inside the macromolecular protein, thereby reducing the L* values. Furthermore, the decrease in the L*, a* and b* color parameters might be due to the destruction of pigment components by US, and this change turned out to be closely intertwined with US power and time [25].

The moisture content of EWPP was closely related to the agglomeration degree during storage, which greatly affects powder dispersion and wetting [26]. As shown in Table 1, the moisture content of the samples was positively correlated with the SL concentration, which may be due to the relatively strong water absorption capacity of the SL, and more SL provided a large contact area with water molecules in the air. Meanwhile, the moisture content of EWPP decreased with increasing US power, indicating that the water was more easily removed by the sound waves passing through the food medium. This was consistent with previous findings that higher power US treatment reduced the moisture content and water activity of rapeseed protein isolate [27].

Packing, storage and transportation costs for powdered products need to consider the bulk density (ρ_bulk_). There are many factors that affect ρ_bulk_, including the powder particle size, moisture content, viscosity and interparticle attraction [28]. Previous studies have proposed that large-size particles should have higher ρ_bulk_ values because the weak frictional force between particles made the powder flow more freely [29]. Furthermore, the contribution of SL concentration to the moisture content and viscosity of EWPP needs to be taken into consideration in this case. The adhesion between particles made it difficult for larger particles to penetrate the space, and the increase in volume made the powder with the same mass obtain smaller ρ_bulk_ values [30]. As expected, the ρ_bulk_ of the powder decreased from 0.48 ± 0.02 g/cm^3^ to 0.32 ± 0.01 g/cm^3^ as the SL concentration increased compared to the control group (Table 1). This may be attributed to the higher moisture content of composite powders at high SL concentrations, where the particles tended to stick together and provided more voids. In addition, the lowest ρ_bulk was_ exhibited at 200 W, which was probably due to the unfolding of protein structures and the formation of the SL-EWPP complex by the samples under US.

### 3.3. Particle Size, Zeta Potential and Free Sulfhydryl (-SH) Group Analysis

**Figure 2:**
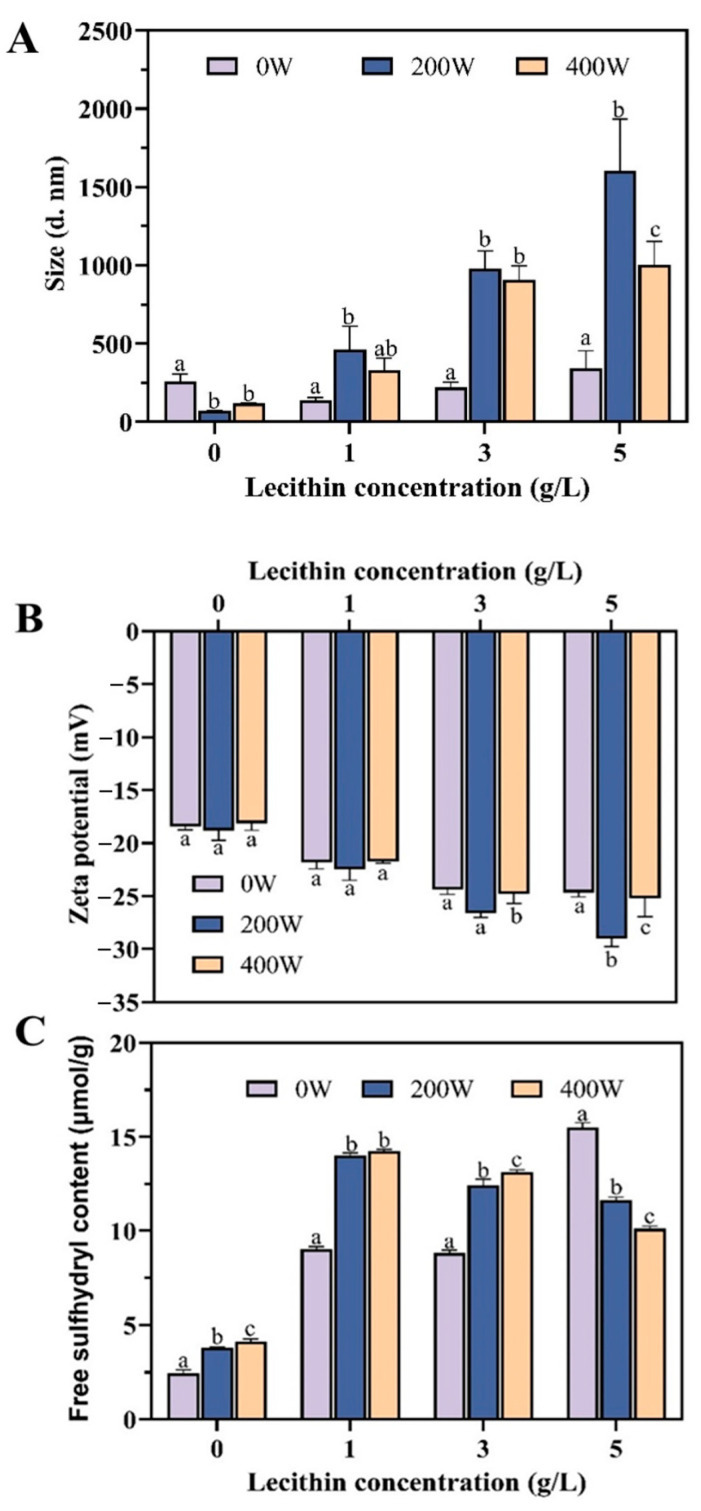
Values of average particle size (**A**), zeta potential (**B**) and free -SH content (**C**) of EWP treated with US-SL. Values that do not bear the same letter are significantly different (*p* < 0.05), and error bars represent the standard deviations.

To further probe the mechanism of US-assisted SL addition to improve the surface properties of SL-EWP, the particle size, zeta potential and free sulfhydryl group content were determined. The effect of SL concentration and US power on the particle size is shown in Figure 2A. Perceptibly, the average particle size of the US was smaller than that of the other groups, and the minimum value (70.56 ± 2.93 nm) occurred at 200 W, which was attributed to the intense fragmentation of particles from the forces of cavitation of US treatment. However, the average particle size value increased to 118.60 ± 4.22 nm at 400 W due to the rearrangement of protein fragments, which did not facilitate the rehydration of EWPP [31]. In general, the average particle size values increased with increasing SL concentration, which was consistent with previous studies that SL and proteins form SL-EWPP complexes through electrostatic and hydrogen bonding interactions [32]. Obviously, the largest average particle size value (1230.41 ± 37.83 nm) was observed at US 200 W-5 g/L SL, which was due to the low-powered US treatment promoting the unfolding of protein structures and providing a large amount of SL binding sites. At the same time, SL formed the micellar structure, which improved the wetting and dispersing properties of EWPP [33]. However, high-powered US treatment resulted in soluble protein aggregation that potentially inhibited the binding of EWP to SL.

In order to further elucidate the internal mechanism of the influence of US-SL treatment on EWPP instant solubility, the zeta potential values of EWPP under different US power and SL concentration were analyzed. The net surface charge of the protein was determined by the ionization of the groups on its surface. As shown in Figure 2B, both the SL addition and US treatment contributed to the exposure of negative charge on the protein surface, which was mainly due to US cavitation and surfactants promoting the loosening and expansion of the protein structure without causing extensive protein aggregation, thus exposing more negative charges [34]. On the other hand, the negatively charged groups in SL were able to neutralize the positively charged amino groups on the surface of EWPP [35]. However, with the increasing of US power, the net negative charge on the protein surface increased first and then decreased. This phenomenon was due to protein aggregation at high-power US conditions, resulting in the negative charge being buried on the protein surface. Notably, the sample with US-assisted SL addition had larger absolute potential values than that of any other conditions, indicating a strong electrostatic repulsion between protein molecules. Admittedly, the increase in electrostatic repulsion could effectively prevent the agglomeration of powders at the air–water interface and in the solution, resulting in a rapidly dispersing solution [36]. It reinforced the fact that US-assisted SL addition facilitated the rehydration of EWPP.

The free -SH groups content was measured to evaluate the influence of US-assisted SL addition on covalent interactions and EWP structure. For the control group, the content of free -SH increased significantly after US treatment (Figure 2C). It is noteworthy that a synergistic effect of low SL concentration (1–3 g/L) and US treatment on the increase in free -SH content was observed, which may be attributed to the protein structure unfolding and disulfide bond breaking, exposing the -SH to the EWPP molecular surface. The variations in the protein structure and turbulence effect under US made the action site originally buried in the EWPP closer to the SL and raised opportunities of collision between them. The hydrophilicity of the protein was enhanced by the combination of EWP and surfactant SL, contributing to the improvement of EWPP rehydration. However, the content of free -SH was negatively correlated with the US power at 5 g/L SL concentration. According to the report of Huang et al. [37], the covalent binding of SL and EWP at high-power US caused protein aggregation. As expected, the results were consistent with the wettability and dispersion of EWPP above.

### 3.4. Surface Hydrophobicity (H_0_)

**Figure 3:**
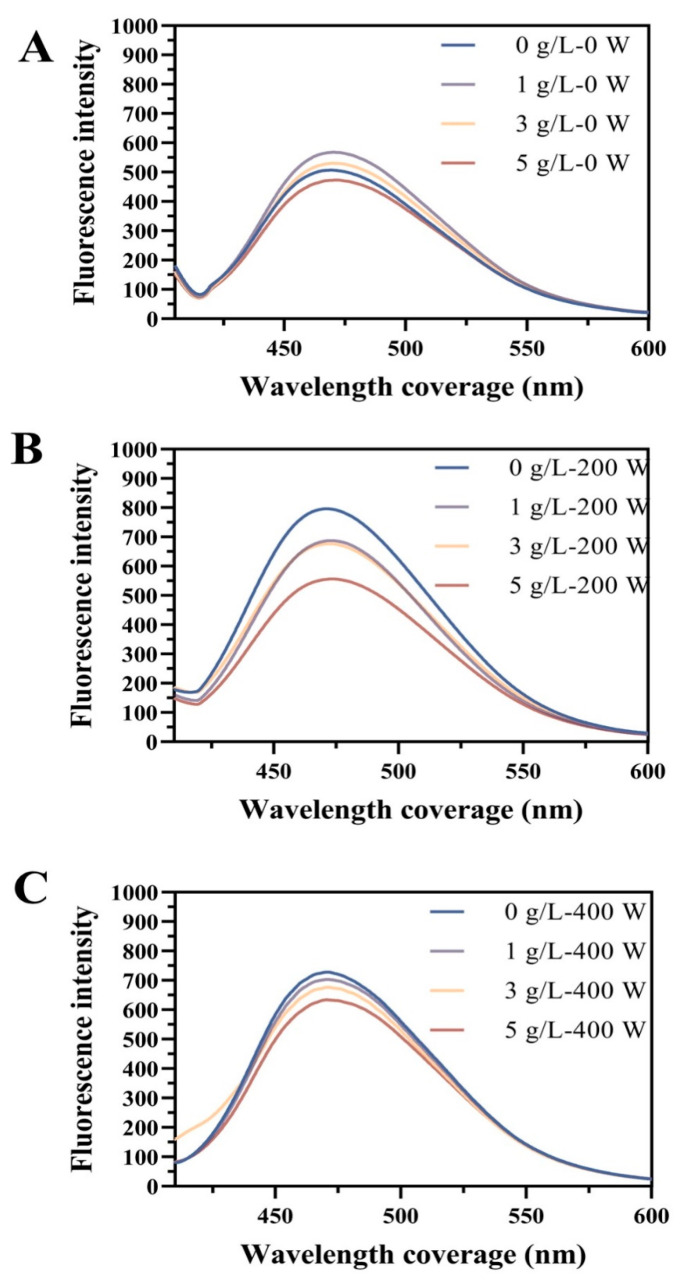
Relative fluorescence intensity of EWP under different SL concentrations (0, 1, 3, 5 g/L) with ultrasound power at (**A**) 0 W, (**B**) 200 W, and (**C**) 400 W.

To explore the influence of rehydration of EWPP and US-assisted SL addition on the conformational changes, the surface hydrophobicity (*H*_0_) was calculated. As displayed in Figure 3, the EWPP fluorescence intensity decreased as the SL concentration increased, which may be due to the hydrophobic interactions between phosphatidylcholine and globulins easily occurring [38]. In this case, SL masked the hydrophobic amino acids on the surface of the EWP and formed the SL-EWP complexes. In addition, the exposure of a large number of hydrophilic phosphate groups on the protein surface may be responsible for the increased wettability and dispersibility of EWPP [39]. Compared with the samples adding SL only (Figure 3A), US-assisted SL addition increased the *H*_0_ of the EWPP. Dramatically, the samples showed the highest fluorescence intensity at 200 W (Figure 3B). These results may be due to the fact that low-powered US induced an unfolding of the EWP structure and provided sufficient hydrophobic binding sites at the same SL concentration. According to reports by Hou et al. [40], the cavitation and mechanical effects of US increased the binding efficiency between EWPP and SL molecules, which was in line with the particle size results in Figure 2A. However, the protein aggregated at US 400 W, which was similar to the results obtained by Jiang et al. [41] that high-powered US protected the hydrophobic region of the protein.

### 3.5. Apparent Viscosity

**Figure 4:**
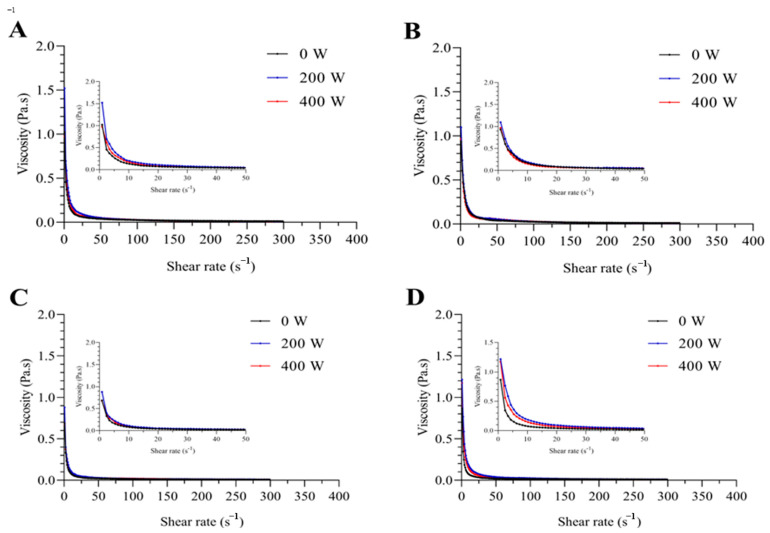
The viscosity of EWP solution under US-SL treatment: (**A**) 0 g/L, (**B**) 1 g/L, (**C**) 3 g/L, (**D**) 5 g/L.

Viscosity changes treated with US-SL were measured to elucidate the rheological behavior of the EWP solutions, which was critical for consumer sensory evaluation of the product. The apparent viscosity of samples as a function of shear rate was reflected in the viscosity rheology curves. As shown in Figure 4, at all concentrations of SL, the solution viscosity increased at first and then decreased as the US power increased, and the maximum was obtained at 5 g/L SL addition. Previous reports suggested that the viscosity of the emulsion depended on the interaction of the protein chain and the changes in the protein structure [42]. Therefore, the unfolding and denaturation of EWPP under 200 W US power was probably the reason for the increase in the EWP solution viscosity. On the other hand, according to the reports of Zisu et al. [43], there was a strong correlation between the emulsion viscosity and protein particle size distribution; thus, the increase in particle size was conducive to the increase in viscosity. Therefore, according to the previous particle size results, it was considered that the viscosity of the EWP solution was significantly improved at the ultrasound power of 200 W due to the formation of hydrophobic interaction and the increase in particle size.

### 3.6. FT-IR and DSC Analysis

**Figure 5:**
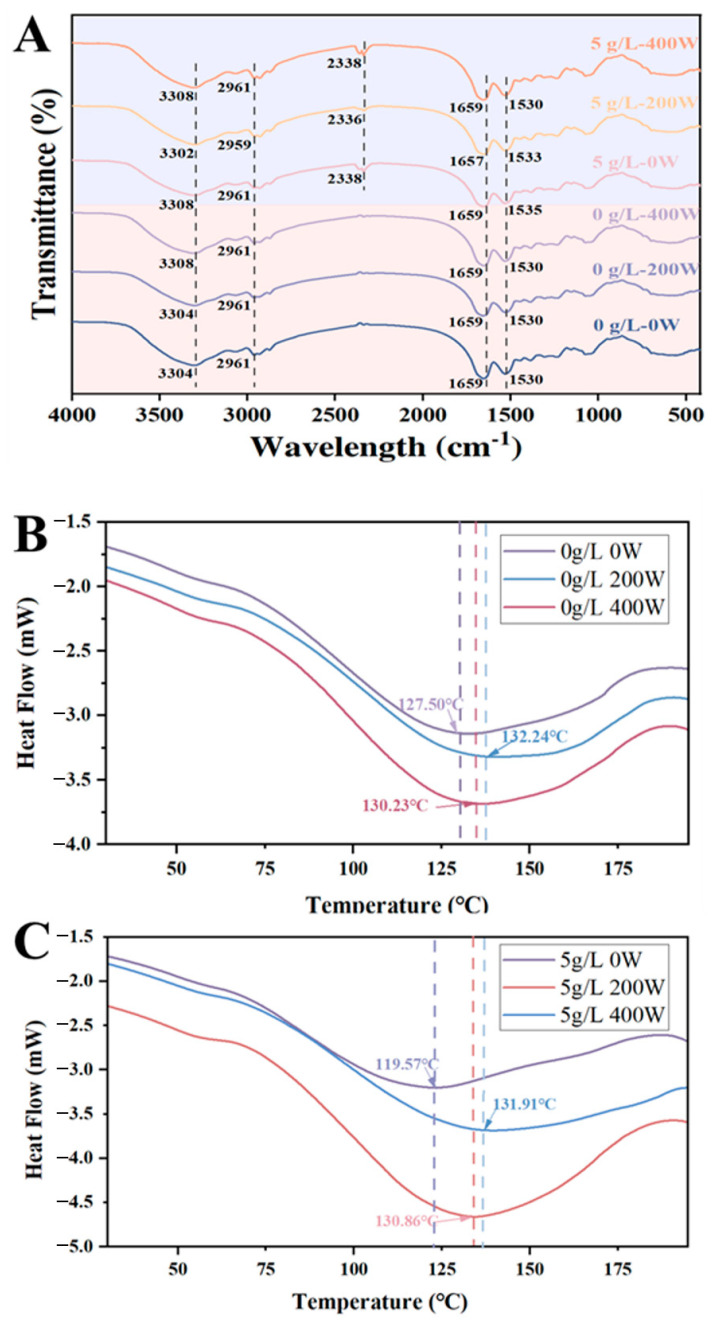
FT-IR spectra (**A**) and DSC curves of EWP with (**B**) single US treatment and (**C**) US-5 g/L SL treatment.

The structures of SL-EWPP samples treated by different US power were determined by FT-IR, as shown in Figure 5A. The typical broad band (amide A) at 3292 cm^−1^ became narrower as the US power increased, indicating that the intermolecular hydrogen bond O-H and N-H groups underwent tensile vibration under ultrasonic action [44]. In addition, we observed enhanced characteristic peaks of the antisymmetric stretching vibration of -CH_2_ for both the single SL-EWPP sample and US-assisted SL-EWPP samples—especially the peak signal intensity at 2960 cm^−1^ and 2927 cm^−1^. This indicated changes in the side chain vibrations and protein structure, thus potentially exposing more hydrophobic groups and charges to alter the surface properties of the EWP [45]. These results suggested that US could destroy the secondary structure of the protein and promote the coupling of EWPP and SL.

The DSC curves of the SL-EWP samples are displayed in Figure 5B. The peak heat absorption of each sample ranged from 100 to 175 °C. The initial two thermal denaturation temperatures (Tp) without US treatment were measured to be 127.50 °C and 119.57 °C, respectively. As the US power increased, the two groups increased to 132.24 °C and 130.86 °C, respectively. It is noteworthy that the ΔH of the samples increased significantly after treatment with 5 g/L SL. These results suggested that EWP with US (200 W)-assisted SL (5 g/L) addition led to proteins with higher thermal stability, which was consistent with the conclusion drawn above.

### 3.7. Scanning Electron Microscope (SEM)

**Figure 6:**
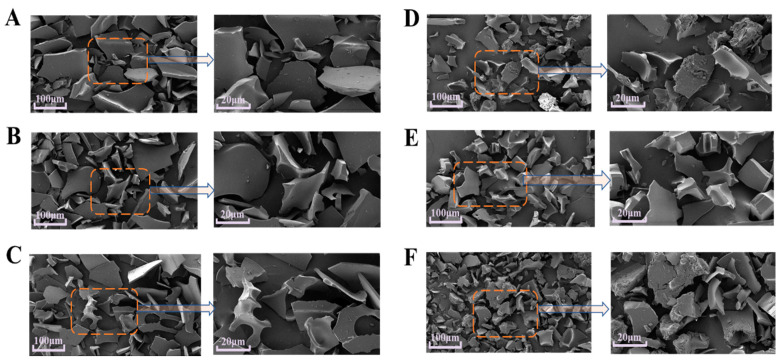
Effect of US-SL treatment on EWPP microscopic morphology. (**A**) Untreated EWPP, (**B**) EWPP with US 200 W-0 g/L SL treatment, (**C**) EWPP with US 400 W-0 g/L SL treatment, (**D**) EWPP with US 0 W-5 g/L SL treatment, (**E**) EWPP with US 200 W-5 g/L SL treatment, (**F**) EWPP with US 400 W-5 g/L SL treatment.

The effect of ultrasound-assisted SL addition on the morphology of EWPP particles was further evaluated using SEM. For the control group, the particles appeared to be uniformly distributed. Interestingly, despite a small amount of aggregation of the particles after 200 W US treatment, the height and diameter were significantly reduced in the SEM images, which was attributed to a disruption in the EWP structure after US treatment, such as covalent bond breaking, low molecular weight peptide generation, and the fragmentation of large aggregates into smaller particles. As the US power increased, the aggregated particles of EWP increased in size, and the 3D microstructure became coarse. Similar results were observed in quinoa proteins, suggesting that high-power sonication reassembles dissociated subunits into protein aggregates, which was consistent with previous particle size results (Figure 6A). It was noteworthy that although previous studies have shown that composite powders containing both SL and EWPP can promote the dispersion of EWPP during solubilization through the interfacial properties of SL (Zhang et al., 2022), a single treatment of SL had no significant effect on the particle size when compared to untreated samples, whereas the addition of SL with the assistance of sonication resulted in larger and more aggregated particles. This phenomenon was attributed to the US-promoted formation of SL-EWP complexes, which largely supported the mechanism of improved EWPP rehydration by US-SL treatment. The best rehydration effect was observed at 200 W US power and 5 g/L SL concentration, which was due to the low US promoting the unfolding of EWPP and providing binding sites for SL. Meanwhile, the coarse and small granularity in the image of EWPP was attributed to the self-agglomeration of EWPP under high power US-SL treatment, which limited its non-covalent interaction with SL.

## 4. Conclusions

In this study, the effect of US-SL treatment on the rehydration behavior of EWPP was evaluated by monitoring the wetting time and dispersing time of the powder. The results showed that US-SL treatment could significantly reduce the wetting and dispersion time of EWPP, which may be due to the combined treatment improving the net negative charge and particle size of the powders, resulting in enlarged pores between the particles and increased intermolecular repulsion, and thus facilitating water infiltration into the powders. However, excessive ultrasonic treatment resulted in the self-aggregation of some soluble protein molecules, which was not conducive to wetting and dispersion. The free -SH group content and *H*_0_ increasing indicated the unfolding of the protein structure, allowing SL to acquire more binding sites on the EWP surface. Additionally, FT-IR and SEM results further confirmed the formation of SL-EWP complexes, thus obtaining better rehydration performance. In short, through the combined treatment of US and SL addition, the instant solubility of EWPP was further improved, and the difference of the influence of US and SL concentrations on the instant solubility was explored. Overall, this work provided a new perspective for the production of instant EWPP products with certain functional and good sensory properties in the food industry.

## Figures and Tables

**Table 1:** The color, moisture content and bulk density of EWPP with US-SL treatment.

Powder		L*	a*	b*	Moisture Content (%)	ρ_bluk_
0 W	0 g/L	105.00 ± 0.26 ^a^	−0.33 ± 0.07 ^a^	17.61 ± 0.05 ^a^	2.39 ± 1.47 ^a^	0.48 ± 0.02 ^a^
	1 g/L	102.27 ± 0.06 ^b^	−1.75 ± 0.09 ^b^	12.45 ± 0.02 ^b^	3.69 ± 2.50 ^b^	0.37 ± 0.02 ^b^
	3 g/L	102.03 ± 0.12 ^c^	−1.63 ± 0.06 ^b^	15.65 ± 0.01 ^c^	3.89 ± 1.03 ^b^	0.35 ± 0.01 ^b^
	5 g/L	98.58 ± 0.22 ^c^	−1.46 ± 0.07 ^c^	14.50 ± 0.01 ^d^	5.89 ± 2.57 ^c^	0.32 ± 0.01 ^c^
200 W	0 g/L	101.90 ± 0.10 ^a^	−2.11 ± 0.02 ^a^	16.82 ± 0.03 ^a^	2.35 ± 1.20 ^a^	0.31 ± 0.01 ^a^
	1 g/L	101.67 ± 0.31 ^a^	−1.81 ± 0.08 ^b^	15.03 ± 0.04 ^b^	3.15 ± 2.12 ^a^	0.29 ± 0.01 ^b^
	3 g/L	100.37 ± 0.15 ^b^	−1.85 ± 0.02 ^b^	17.24 ± 0.01 ^c^	4.27 ± 1.84 ^b^	0.27 ± 0.01 ^c^
	5 g/L	99.90 ± 0.04 ^c^	−0.94 ± 0.03 ^c^	13.44 ± 0.03 ^d^	4.31 ± 0.52 ^b^	0.27 ± 0.01 ^c^
400 W	0 g/L	101.20 ± 0.10 ^a^	−2.06 ± 0.12 ^a^	15.16 ± 0.06 ^a^	2.07 ± 2.21 ^a^	0.39 ± 0.01 ^a^
	1 g/L	101.20 ± 0.10 ^a^	−1.92 ± 0.03 ^ab^	14.27 ± 0.02 ^b^	1.60 ± 1.74 ^a^	0.37 ± 0.01 ^b^
	3 g/L	101.47 ± 0.06 ^b^	−1.78 ± 0.09 ^b^	12.15 ± 0.02 ^c^	3.35 ± 0.19 ^a^	0.37 ± 0.01 ^b^
	5 g/L	99.14 ± 0.12 ^c^	−1.31 ± 0.05 ^c^	14.32 ± 0.05 ^b^	2.60 ± 1.75 ^a^	0.36 ± 0.01 ^b^

Means with different superscripted letters in the same column are significantly different (*p* < 0.05).

## Data Availability

The original contributions presented in the study are included in the article, further inquiries can be directed to the corresponding author.

## References

[1] Dong X., Zhang Y.-Q. (2021). An insight on egg white: From most common functional food to biomaterial application. J. Biomed. Mater. Res. Part B-Appl. Biomater..

[2] Li P., Jin Y., Sheng L. (2020). Impact of microwave assisted phosphorylation on the physicochemistry and rehydration behaviour of egg white powder. Food Hydrocoll..

[3] Liu Y., Huang C., Wang J., Li Z., Xu Q., Chen L., Feng X., Ma M. (2022). Improving rehydration of egg white powder through modifying its physicochemistry properties by ultrasound-assisted glutaminase deamidation. Food Hydrocoll..

[4] Deng L. (2021). Current Progress in the Utilization of Soy-Based Emulsifiers in Food Applications—A Review. Foods.

[5] Ji J., Cronin K., Fitzpatrick J., Miao S. (2017). Enhanced wetting behaviours of whey protein isolate powder: The different effects of lecithin addition by fluidised bed agglomeration and coating processes. Food Hydrocoll..

[6] Lallbeeharry P., Tian Y., Fu N., Wu W., Woo M., Selomulya C., Chen X. (2014). Effects of ionic and nonionic surfactants on milk shell wettability during co-spray-drying of whole milk particles. J. Dairy Sci..

[7] Ji J., Cronin K., Fitzpatrick J., Fenelon M., Miao S. (2015). Effects of fluid bed agglomeration on the structure modification and reconstitution behaviour of milk protein isolate powders. J. Food Eng..

[8] Liu X., Yang Q., Yang M., Du Z., Wei C., Zhang T., Liu B., Liu J. (2021). Ultrasound-assisted Maillard reaction of Ovalbumin/Xylose:the enhancement of functional properties and its mechanism. Ultrason. Sonochemistry.

[9] Qu W., Zhang X., Chen W., Wang Z., He R., Ma H. (2018). Effects of ultrasonic and graft treatments on grafting degree, structure, functionality, and digestibility of rapeseed protein isolate-dextran conjugates. Ultrason. Sonochemistry.

[10] Tian Y., Fu N., Wu W., Zhu D., Huang J., Yun S., Chen X. (2014). Effects of Co-spray Drying of Surfactants with High Solids Milk on Milk Powder Wettability. Food Bioprocess Technol..

[11] Zhang Y., Hua Y., Kong X. (2008). Effects of limited enzymatic hydrolysis on dispersion properties of soy protein concentrate. Cereals Oils.

[12] Zhang T., Yang Y., Zhang M., Jiang H., Yan Z., Liu J., Liu X. (2022). Effect of soy lecithin concentration on physiochemical properties and rehydration behavior of egg white protein powder: Role of dry and wet mixing. J. Food Eng..

[13] Masum A., Huppertz T., Chandrapala J., Adhikari B., Zisu B. (2020). Physicochemical properties of spray-dried model infant milk formula powders: Influence of whey protein-to-casein ratio. Int. Dairy J..

[14] Erdem B., Kaya S. (2021). Production and application of freeze dried biocomposite coating powders from sunflower oil and soy protein or whey protein isolates. Food Chem..

[15] Kapoor R., Feng H. (2022). Characterization of physicochemical, packing and microstructural properties of beet, blueberry, carrot and cranberry powders: The effect of drying methods. Powder Technol..

[16] Liu J., Jiang H., Zhang M., Gong P., Yang M., Zhang T., Liu X. (2022). Ions-regulated aggregation kinetics for egg white protein: A promising formulation with controlled gelation and rheological properties. Int. J. Biol. Macromol..

[17] Zhang T., Zhang M., Gong P., Jiang H., Liu J., Liu X. (2022). Ions-induced ovalbumin foaming properties enhancement: Structural, rheological, and molecular aggregation mechanism. Food Hydrocoll..

[18] Shimada K., Cheftel J. (1988). Determination of sulfhydryl groups and disulfide bonds in heat-induced gels of soy protein isolate. J. Agric. Food Chem..

[19] Zhang Y., Liu J., Yan Z., Zhang R., Du Z., Shang X., Zhang T., Liu X. (2024). Mechanism of ultrasound-induced soybean/egg white composite gelation: Gel properties, morphological structure and co-aggregation kinetics. Int. J. Biol. Macromol..

[20] Yan Z., Liu J., Ren J., Li C., Wang Z., Dai L., Cao S., Zhang R., Liu X. (2023). Magnesium ions regulated ovalbumin-lysozyme heteroprotein complex: Aggregation kinetics, thermodynamics and morphologic structure. Int. J. Biol. Macromol..

[21] Zhang T., Li S., Yang M., Li Y., Ma S., Zhang H., Li L., Liu X., Liu J., Du Z. (2024). The influence of unique interfacial networks based on egg white proteins for the stabilization of high internal phase Pickering emulsions: Physical stability and free fatty acid release kinetics. Food Chem..

[22] Yao K., Xia Y., Gao H., Chen W., Hou J., Jiang Z. (2019). Influence of Ultrasonic Power and Ultrasonic Time on the Physicochemical and Functional Properties of Whey Protein Isolate. Int. J. Food Eng..

[23] Jin H., Sun S., Sun Z., Wang Q., Jin Y., Sheng L. (2022). Ultrasonic-assisted spray drying as a tool for improving the instant properties of egg white powder. Food Struct..

[24] Zhou B., Zhang M., Fang Z., Liu Y. (2015). Effects of ultrasound and microwave pretreatments on the ultrafiltration desalination of salted duck egg white protein. Food Bioprod. Process..

[25] Thalía Flores-Jiménez N., Armando Ulloa J., Esmeralda Urías-Silvas J., Carmen Ramírez-Ramírez J., Ulises Bautista-Rosales P., Gutiérrez-Leyva R. (2022). Influence of high-intensity ultrasound on physicochemical and functional properties of a guamuchil *Pithecellobium dulce* (Roxb.) seed protein isolate. Ultrason. Sonochemistry.

[26] Jung H., Lee Y., Yoon W. (2018). Effect of Moisture Content on the Grinding Process and Powder Properties in Food: A Review. Processes.

[27] Flores-Jimenez N., Ulloa J., Silvas J., Ramirez J., Ulloa P., Rosales P., Carrillo Y., Leyva R. (2019). Effect of high-intensity ultrasound on the compositional, physicochemical, biochemical, functional and structural properties of canola (*Brassica napus* L.) protein isolate. Food Res. Int..

[28] Yuliana M., Truong C., Huynh L., Ho Q., Ju Y. (2014). Isolation and characterization of protein isolated from defatted cashew nut shell: Influence of pH and NaCl on solubility and functional properties. LWT Food Sci. Technol..

[29] Jinapong N., Suphantharika M., Jamnong P. (2008). Production of instant soymilk powders by ultrafiltration, spray drying and fluidized bed agglomeration. J. Food Eng..

[30] Teo A., Lam Y., Lee S., Goh K. (2021). Spray drying of whey protein stabilized nanoemulsions containing different wall materials—Maltodextrin or trehalose. LWT-Food Sci. Technol..

[31] Ma C., Jiang W., Chen G., Wang Q., McClements D., Liu X., Liu F., Ngai T. (2021). Sonochemical effects on formation and emulsifying properties of zein-gum Arabic complexes. Food Hydrocoll..

[32] Sun X., Wang C., Guo M. (2018). Interactions between whey protein or polymerized whey protein and soybean lecithin in model system. J. Dairy Sci..

[33] Otzen D. (2011). Protein–surfactant interactions: A tale of many states. Biochim. et Biophys. Acta (BBA) Proteins Proteom..

[34] Zou H., Zhao N., Li S., Sun S., Dong X., Yu C. (2020). Physicochemical and emulsifying properties of mussel water-soluble proteins as affected by lecithin concentration. Int. J. Biol. Macromol..

[35] Surh J., Decker E., McClements D. (2005). Influence of pH and pectin type on properties and stability of sodium-caseinate stabilized oil-in-water emulsions. Food Hydrocoll..

[36] Gao W., Jiang Z., Du X., Zhang F., Liu Y., Bai X., Sun G. (2020). Impact of Surfactants on Nanoemulsions based on Fractionated Coconut Oil: Emulsification Stability and in vitro Digestion. J. Oleo Sci..

[37] Huang L., Zhang W., Yan D., Ma L., Ma H. (2020). Solubility and aggregation of soy protein isolate induced by different ionic liquids with the assistance of ultrasound. Int. J. Biol. Macromol..

[38] Li J., Li Y., Guo S. (2014). Association of phosphatidylcholine with soybean 11S globulin. Food Sci. Biotechnol..

[39] Shen Y., Chang C., Shi M., Su Y., Gu L., Li J., Yang Y. (2020). Interactions between lecithin and yolk granule and their influence on the emulsifying properties. Food Hydrocoll..

[40] Hou F., He L., Ma X., Wang D., Ding T., Ye X., Liu D. (2020). Ultrasound enhanced the binding ability of chitinase onto chitin: From an AFM insight. Ultrason. Sonochemistry.

[41] Jiang L., Wang J., Li Y., Wang Z., Liang J., Wang R., Chen Y., Ma W., Qi B., Zhang M. (2014). Effects of ultrasound on the structure and physical properties of black bean protein isolates. Food Res. Int..

[42] Westphalen A., Briggs J., Lonergan S. (2006). Influence of muscle type on rheological properties of porcine myofibrillar protein during heat-induced gelation. Meat Sci..

[43] Zisu B., Bhaskaracharya R., Kentish S., Ashokkumar M. (2010). Ultrasonic processing of dairy systems in large scale reactors. Ultrason. Sonochemistry.

[44] Chen X., Zhou R., Xu X., Zhou G., Liu D. (2017). Structural modification by high-pressure homogenization for improved functional properties of freeze-dried myofibrillar proteins powder. Food Res. Int..

[45] Li Q., Zhang X., Tang S., Mi S., Lu L., Zeng Q., Xia M., Cai Z. (2022). Improved effect of ultrasound-assisted enzymolysis on egg yolk powder: Structural properties, hydration properties and stability characteristics. Food Chem..

